# M3: multimodal artificial intelligence for medical report generation and visual question answering from 3D abdominal CT scans

**DOI:** 10.1093/bjrai/ubaf011

**Published:** 2025-07-17

**Authors:** Abdullah Hosseini, Ahmed Ibrahim, Ahmed Serag

**Affiliations:** AI Innovation Lab, Weill Cornell Medicine—Qatar, Doha, 24144, Qatar; AI Innovation Lab, Weill Cornell Medicine—Qatar, Doha, 24144, Qatar; AI Innovation Lab, Weill Cornell Medicine—Qatar, Doha, 24144, Qatar

**Keywords:** artificial intelligence, multimodal analysis, vision-language models, medical report generation, 3D images

## Abstract

**Objectives:**

Medical imaging is indispensable for diagnosis, with abdominal imaging playing a pivotal role in generating medical reports and informing clinical decision-making. Recent works in artificial intelligence (AI), particularly in multimodal approaches such as vision-language models, have demonstrated significant potential to enhance medical image analysis by seamlessly integrating visual and textual data. While 2D imaging has been the main focus of many studies, the enhanced spatial detail and volumetric consistency offered by 3D images, such as CT scans, remain relatively underexplored. This gap underscores the need for innovative approaches to unlock the potential of 3D imaging in clinical workflows.

**Methods:**

In this study, we utilized a multimodal AI pipeline, Phi3-V, to address 2 key challenges in abdominal imaging: generating clinically coherent medical reports from 3D CT images and performing visual question answering based on these images.

**Results:**

Our optimized model attained an average GREEN score of 0.409 for medical report generation and an accuracy of 79% for multiple-choice visual question answering on the validation cases.

**Conclusions:**

These findings demonstrate the potential of multimodal AI in advancing the analysis of 3D medical imaging, paving the way for more robust and efficient applications in healthcare.

**Advances in knowledge:**

This study advances the use of multimodal AI for 3D CT imaging, achieving improvements in medical report generation and visual question answering.

## Introduction

Nowadays, artificial intelligence (AI) has propelled marked improvements in medical image analysis, improving both the speed and accuracy of interpretation.^1^^,^^2^ These advancements have already transformed many aspects of diagnostic workflows, reducing human error and improving efficiency.^3–5^ By bridging the gap between imaging and textual data, a multimodal approach offers the potential for deeper insights into patient health, paving the way for more precise and comprehensive clinical decision-making. Furthermore, it can support the development of robust AI systems capable of addressing challenges in personalized medicine and advanced diagnostics.

Recent progress in vision-language models (VLMs), particularly their application to 2D medical imaging,^6^^,^^7^ demonstrates that overcoming longstanding challenges is no longer a pipe dream. Early approaches to medical report generation (MRG) often relied on CNN-RNN hybrid architectures. More recent transformer-based models, such as METransformer^8^ and KiUT,^9^ have introduced multi-expert token mechanisms and incorporated knowledge graphs to improve diagnostic alignment and reduce hallucinations.

With the advent of large language models (LLMs), newer methods like R2GenGPT^10^ and ClinicalBLIP^11^ have integrated vision-language alignment modules to map visual features from X-rays into LLM embedding spaces. VLMs such as XrayGPT^12^ and CXR-LLAVA^13^ have further narrowed the modality gap by fine-tuning pretrained architectures on clinical datasets, leveraging components like Q-Former to align image tokens with textual prompts and domain-specific medical knowledge.

Extending vision-language capabilities to 3D imaging holds the potential to revolutionize how medical data is processed and interpreted. However, analysing of 3D data introduces critical challenges. Unlike 2D images, 3D CT scans require models that can extract spatial features and maintain consistency across volumetric slices. Moreover, the computational demands of processing 3D data significantly increase model complexity and training requirements.^14^

Recent advances in 3D MRG have focused on developing architectures that enhance both diagnostic accuracy and clinical relevance. CT2Rep^15^ introduced a 3D encoder-to-decoder transformer framework for chest CT scans, enabling direct report generation from volumetric inputs. Building on this, MvKeTR^16^ improved performance through a multi-view encoder that processes axial, coronal, and sagittal planes, combined with a cross-modal knowledge retrieval mechanism to enrich semantic alignment. M3D,^17^ in conjunction with LLaMA,^18^ leveraged a CLIP-inspired pretrained 3D encoder with spatial pooling and segmentation capabilities to support a comprehensive understanding of 3D medical images. Extending this line of work, 3D-CT-GPT^19^ refined the CT encoder by incorporating CT-ViT, 3D pooling, and projection layers, leading to further performance improvements.

In this study, we propose a framework that combines a vision encoder with Phi3-V,^20^ a compact language model, to address the challenges of 3D CT scan analysis. By integrating the spatial reasoning capabilities of VLMs with the contextual understanding of language models, our method aims to enhance tasks such as MRG and visual question answering (VQA). This approach offers a precise and efficient solution for multimodal AI in abdominal imaging and lays the foundation for future advancements in the automated interpretation of complex medical datasets. The code is available at https://github.com/serag-ai/M3.

## Methods

### Dataset

#### Overview

The AMOS-MM dataset^21^ is a comprehensive resource designed to facilitate multimodal analysis of abdominal imaging, supporting tasks such as MRG, and VQA. The dataset includes medical reports, with a focus on the Findings section across 3 anatomical regions: chest, abdomen, and pelvis. Each report is provided in JSON format, with distinct entries for each region. If a region is not addressed in a specific case, it is represented by an empty string.

The VQA component assesses algorithms on their ability to combine visual and clinical knowledge. Tasks are categorized into 2 groups: *Perception* (eg, anatomical identification, attribute recognition, and quantitative assessment) and *Reasoning* (eg, disease analysis, risk evaluation, and therapeutic suggestion). Each VQA task is presented as a multiple-choice question with a single correct answer, also formatted in JSON format.

#### Dataset preparation and refinement

In analysing the AMOS-MM dataset, a significant imbalance was observed in the distribution of MRG samples, particularly across anatomical regions and report types. Of the 1288 total examples, the dataset includes 1285 abdominal organ reports, 1117 pelvic organ reports, and only 378 chest organ reports. This imbalance negatively affects model performance for chest organ report generation.

Additionally, several samples contain notably shorter captions, often limited to a single sentence, which introduces inconsistency in training and results in models generating shorter outputs during evaluation. To address these challenges, we created 3 refined versions of the dataset, each tailored for fine-tuning MRG models:

##### Version 1

To address the issue of dataset imbalance in MRG, we incorporated additional data from the CtRate dataset,^15^^,^^22^ a dataset specifically designed for chest organs. Approximately 2000 samples were incorporated, each containing at least 2 positive abnormalities from the following categories: “Pericardial effusion,” “Lung opacity,” “Pleural effusion,” “Bronchiectasis,” and “Interlobular septal thickening.”

##### Version 2

To tackle the problem of generating short captions, we utilized only the AMOS-MM dataset but removed captions that were shorter (typically 1 sentence) from the training set. This approach aimed to ensure that the model consistently predicts longer sentences.

##### Version 3

This version combines the approaches of version 1 and version 2 to address both issues. Specifically, we removed short sentences from the AMOS-MM dataset and added 2000 samples with specific abnormalities to the dataset from the CtRate dataset.

For VQA, the dataset comprises 13 751 examples, with the distribution of correct answers being as follows: 1249 for option A, 5227 for option B, 4729 for option C, and 2546 for option D. To address the issue of data imbalance, we restructured the dataset by equally distributing the questions across each answer choice, ensuring that the total number of correct responses for each option is almost equal.

### Vision-language model

#### Model architecture

**Figure 1.**
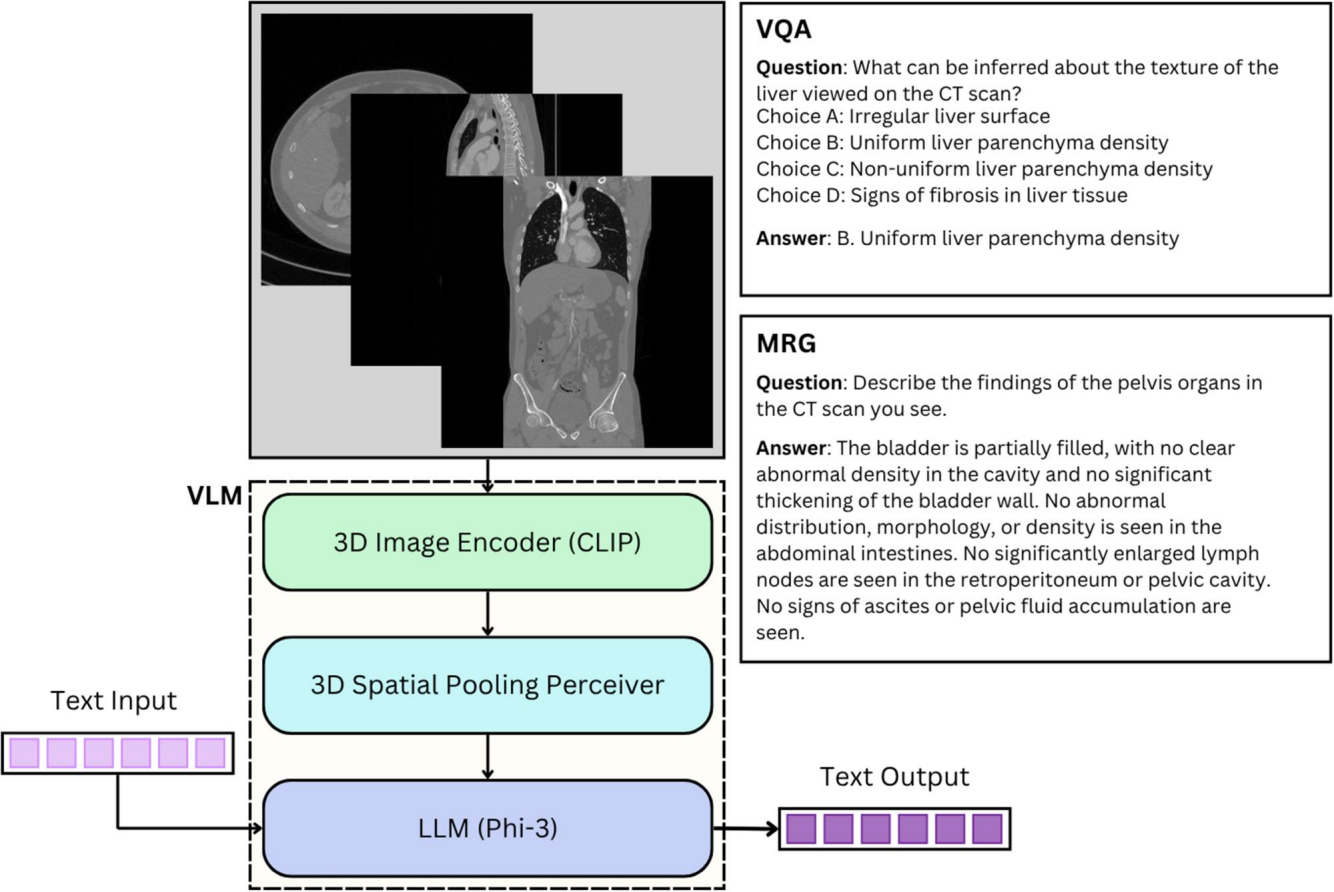
Architecture of the M3 model, illustrating the core modules and data pathways employed for MRG and VQA. The model effectively integrates vision and language streams to facilitate specialized task execution with high efficiency. Abbreviations: LLM = large language model; VLM = vision-language model; MRG = medical report generation; VQA = visual question answering.

In this study, we fine-tuned the Phi3-V^20^ model, which functions as a generalist VLM for 3D medical image analysis. Figure 1 provides an overview of the architecture. The model consists of 3 primary components: (1) language, (2) projector, and (3) vision.

For the vision component, we used the pretrained M3D model^17^ as the foundational image encoder, which was fine-tuned in subsequent stages. The pretrained vision encoder was initially trained using the CLIP approach,^23^ where both 3D CT scans and text were jointly trained. Additionally, to effectively integrate the pretrained CLIP-based image encoder with the language model, we employed a pretrained 3D spatial pooling mechanism from the M3D architecture.

In our approach to language modelling tasks, we utilized 2 different base models: the Phi3 pretrained model and the M3D model. For MRG, we selected the Phi3 pretrained model as the initial weights, while for VQA, we based our work on the M3D model. This selection allowed us to tailor the model choice to the specific requirements of each task.

#### Temperature adjustment in inference

As part of our ablation study, we varied the temperature parameter of the language component in our VLM to examine its influence on the MRG task. This investigation was motivated by preliminary observations suggesting that different temperature settings noticeably affect the quality of the consistency of the generated reports.

Temperature regulates how a language model weights the probabilities of different possible next tokens, influencing the randomness or determinism of its outputs. To modulate the model’s creativity in report generation, the temperature parameter was varied between 0.1 and 0.9, while maintaining a constant top-*P* value. In contrast, for VQA, we utilized the default prediction settings with the temperature fixed at 0.9.

#### Prompt formatting and optimization

For VQA, variations in prompt structure can sometimes enhance the model’s performance in answering questions. To investigate the impact of prompt formatting on the model’s performance in answering multiple-choice questions, we modified the prompt format for the VQA task as part of our ablation study.

To identify the optimal prompt format for the VQA task, we trained the model using the original AMOS-MM dataset and compared its accuracy across different prompt structures. The prompt formats we tested are as follows:

1. {QUESTION}. Correct choice is {ANSWER}.
2. {QUESTION}. {ANSWER}.
3. {QUESTION}. {ANSWER})

**Figure 2.**
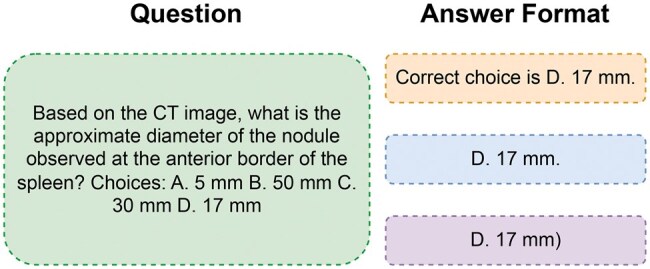
Examples of fine-tuning prompts for Phi3-V on the medical VQA task. All prompts share identical question formats but vary in the answer structure to guide the model’s response style for multiple-choice questions. Abbreviation: VQA = visual question answering.

In this setup, *{QUESTION}* represents the input medical question, while *{ANSWER}* is replaced by the correct choice label (*A, B, C, or D*) from the dataset. However, the model is expected to generate a full explanatory phrase rather than just the letter choice. See Figure 2 for more details.

Additionally, to determine whether the model confuses the answer choice “A” with the article letter “a,” we introduced a fourth prompt format in which the answer “A” was replaced with “X”. This modification allowed us to evaluate whether such an adjustment improved the model’s performance.

### Evaluation metrics

For MRG, we strictly adhered to the challenge’s designated evaluation metrics and utilized the GREEN score (Generative Radiology Report Evaluation and Error Notation).^24^ This recently developed metric is specifically designed for assessing radiology report generation, focusing on the factual correctness and clinical relevance of generated reports. Unlike traditional metrics, the GREEN score leverages a language model to provide a more robust and context-aware evaluation.

It should be noted that the current LLM-based GREEN metric struggles with lengthy reports, as it was trained on shorter ones. To minimize evaluation errors, it is decided to calculate the GREEN score separately for each of the 3 organs (pelvis, chest, abdomen), provided the corresponding ground truth is available. The final score for each organ will be the average of these section-wise scores. In VQA, accuracy is used as the metric to evaluate the proportion of correctly answered questions on a question-wise basis.

### Statistical analysis

To test differences between the results of both models in downstream tasks, *t*-tests were used for normally distributed data, while the non-parametric Mann-Whitney *U* test was applied to non-normal distributions (normality was assessed using the Shapiro-Wilk test). *P* < .05 were considered significant after controlling error using false discovery rate.

## Results

Table 1 summarizes the results for MRG, highlighting the model’s performance across different dataset versions using the GREEN score metric. The comparison includes evaluations on the original AMOS-MM dataset and the refined versions 1, 2, and 3. Experiments were conducted using learning rates of 7 × 10^−4^, 5 × 10^−5^, and 7 × 10^−6^, with training performed over 5 epochs. For clarity, we report only the best results for each combination of dataset version and learning rate.

**Table 1.** Results for the report generation task, comparing performance across different datasets.

Model	Dataset	Epoch	LR	Pelvis	Chest	Abdomen	Average
Vit3D^25^	AMOS-MM	–	–	0.37	0.20	0.32	0.30
Ours	AMOS-MM	5	7e−4	0.476	0.227	0.422	0.375
		5	5e−5	0.362	0.193	0.346	0.301
	Version 1	5	7e−4	0.473	0.245	0.443	0.387
		5	5e−5	0.459	0.261	0.425	0.382
		3	7e−6	**0.516**	0.225	**0.456**	0.399
	Version 2	5	7e−4	0.475	0.208	0.391	0.358
		5	5e−5	0.308	0.151	0.249	0.236
		3	7e−6	0.455	0.231	0.392	0.360
		5	7e−4	0.465	0.183	0.390	0.346
	Version 3	3	5e−5	0.466	**0.282**	0.410	0.386
		5	7e−6	0.511	0.276	0.439	**0.409**

For the sake of conciseness, we do not present the detailed results for all configurations and epochs. Instead, we report only the best-performing model, which achieved the highest average GREEN scores across various dataset versions and learning rates. The highest GREEN score is highlighted in bold, and the second highest is underlined.

Among the different versions of MRG datasets, version 3 achieved the highest average GREEN score (0.409), with notable improvements in performance for both the pelvis and chest regions. Version 1 also showed an increase in average performance (0.399) compared to the original dataset (0.375), attributed to the addition of samples focused on chest abnormalities. In contrast, Version 2, which removed shorter captions, resulted in a lower average score (0.360).

A paired *t*-test comparing the top-performing model (version 3) with other variants revealed no statistically significant difference between version 3 and either the original model (*P* = .068) or version 1 (*P* = .68). However, statistically significant differences were observed between version 3 and both version 2 and Vit3D^25^ (*P* < .05).

**Figure 3.**
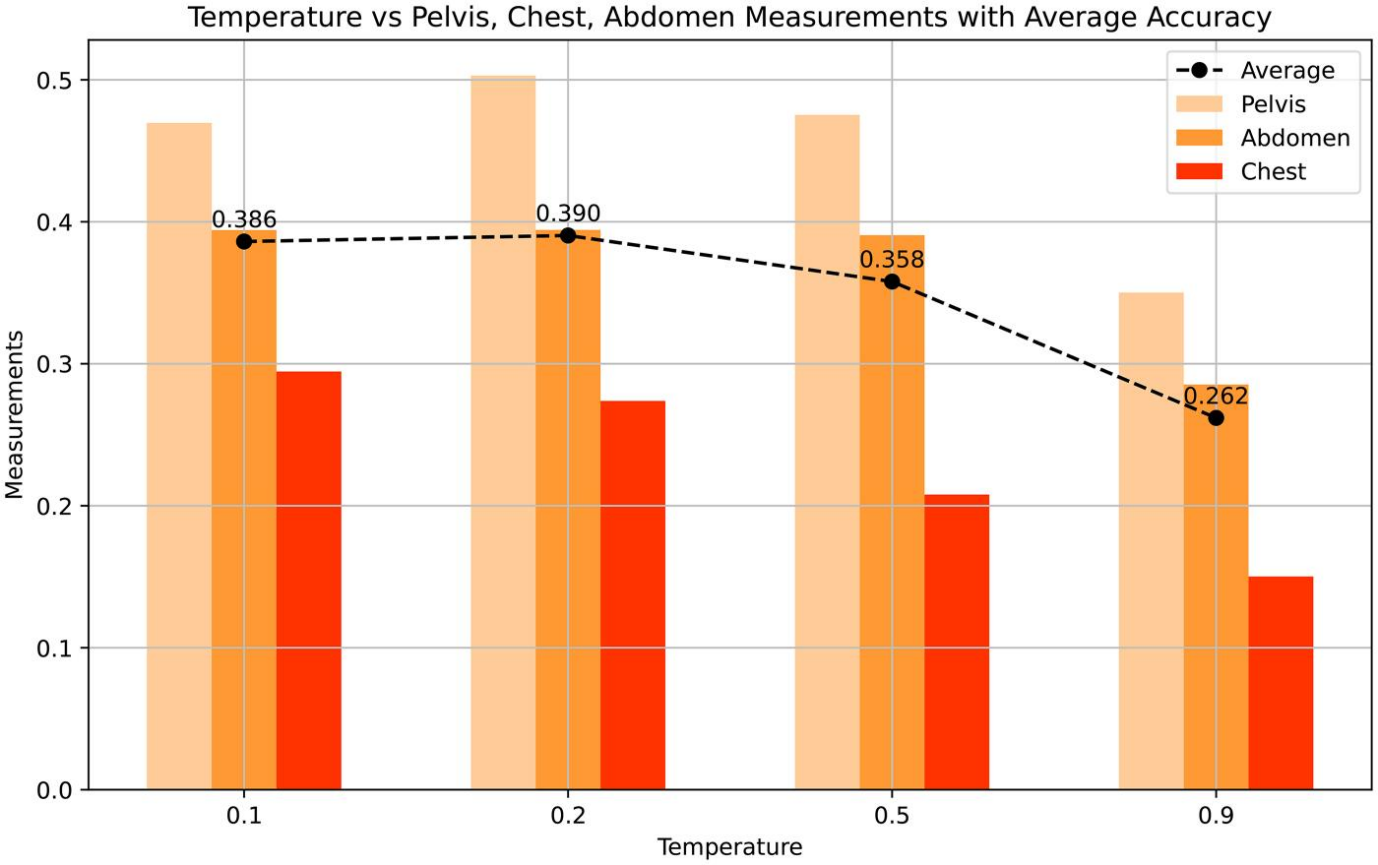
The relationship between temperature and the measurements for the pelvis, chest, and abdomen. Notably, the measurement at temperature 0.2 shows the highest average accuracy of 0.390, indicating better overall performance compared to other temperatures.

**Figure 4.**
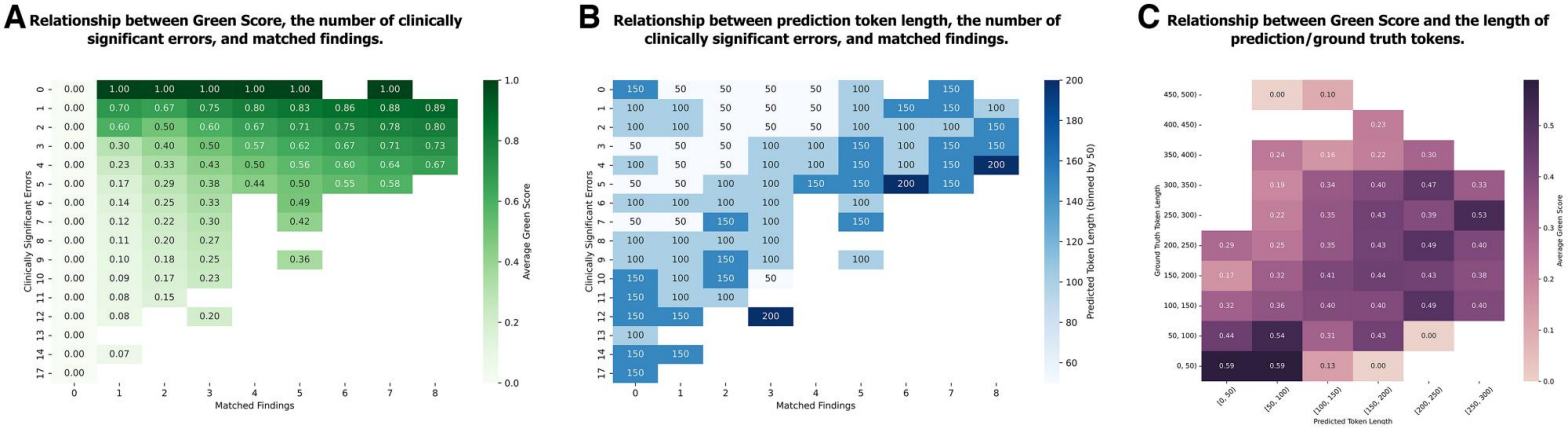
(A) Average GREEN score based on clinically significant errors and matched findings. (B) Average predicted token length associated with clinically significant errors and matched findings. (C) Illustration of the correlation between ground-truth and predicted report token length.

Figure 3 shows the effect of the temperature value on the model’s overall performance score across the pelvis, chest, and abdomen regions. Figure 4 illustrates the relationship between the total number of correct findings, clinically significant errors in the generated reports, the GREEN score, and the predicted token length. Additionally, Figure 3C explores the correlation between the predicted report length and the ground-truth token length with the achieved GREEN score.

**Figure 5.**
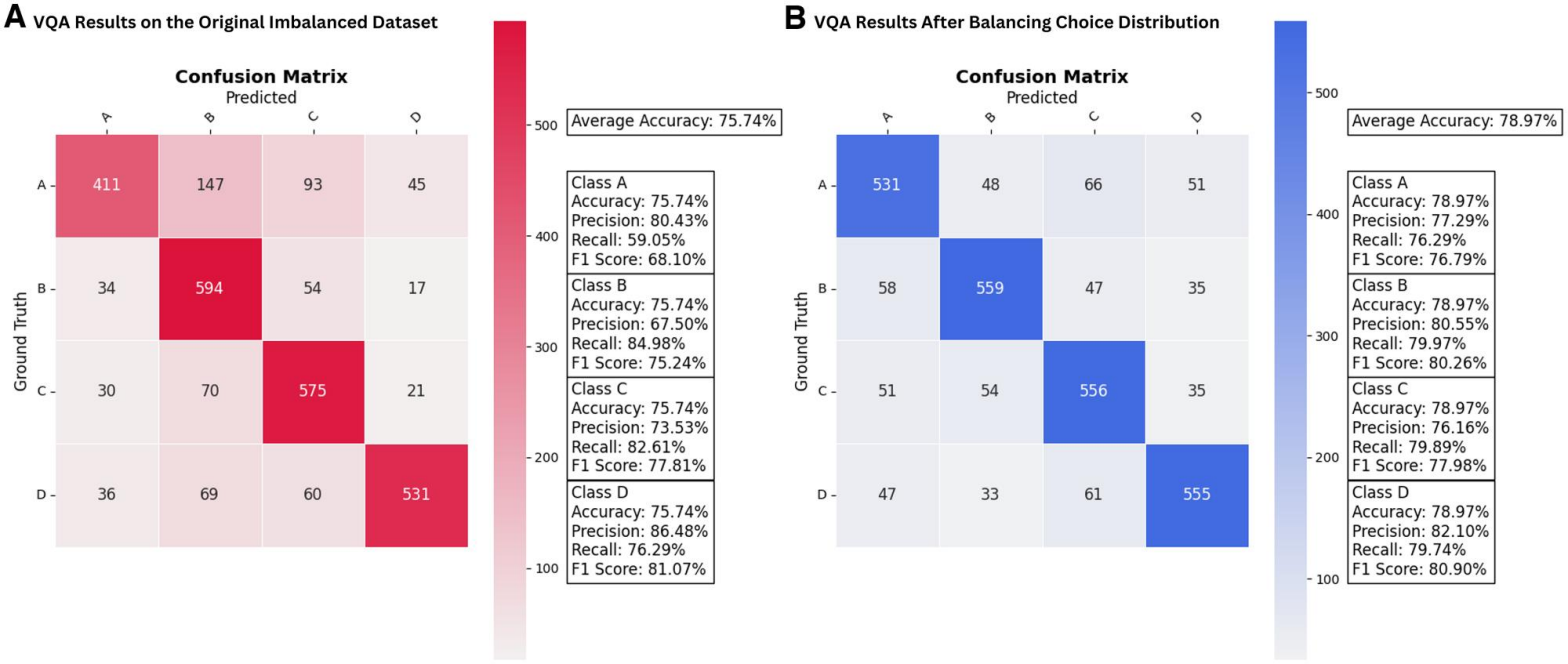
Model trained on (A) an imbalanced dataset and (B) a balanced dataset. As evident in the image, the model successfully predicts most Classes A and D correctly.

Concerning the model’s efficacy in addressing the VQA task, Figure 5 illustrates the model’s performance across both the imbalanced and balanced versions of the dataset for VQA. Although the balanced version demonstrates improved accuracy, particularly for underrepresented answer choices, A *t*-test comparing the F1 scores and accuracies between the imbalanced and balanced versions of the dataset revealed no statistically significant difference (*P* = .101).

**Figure 6.**
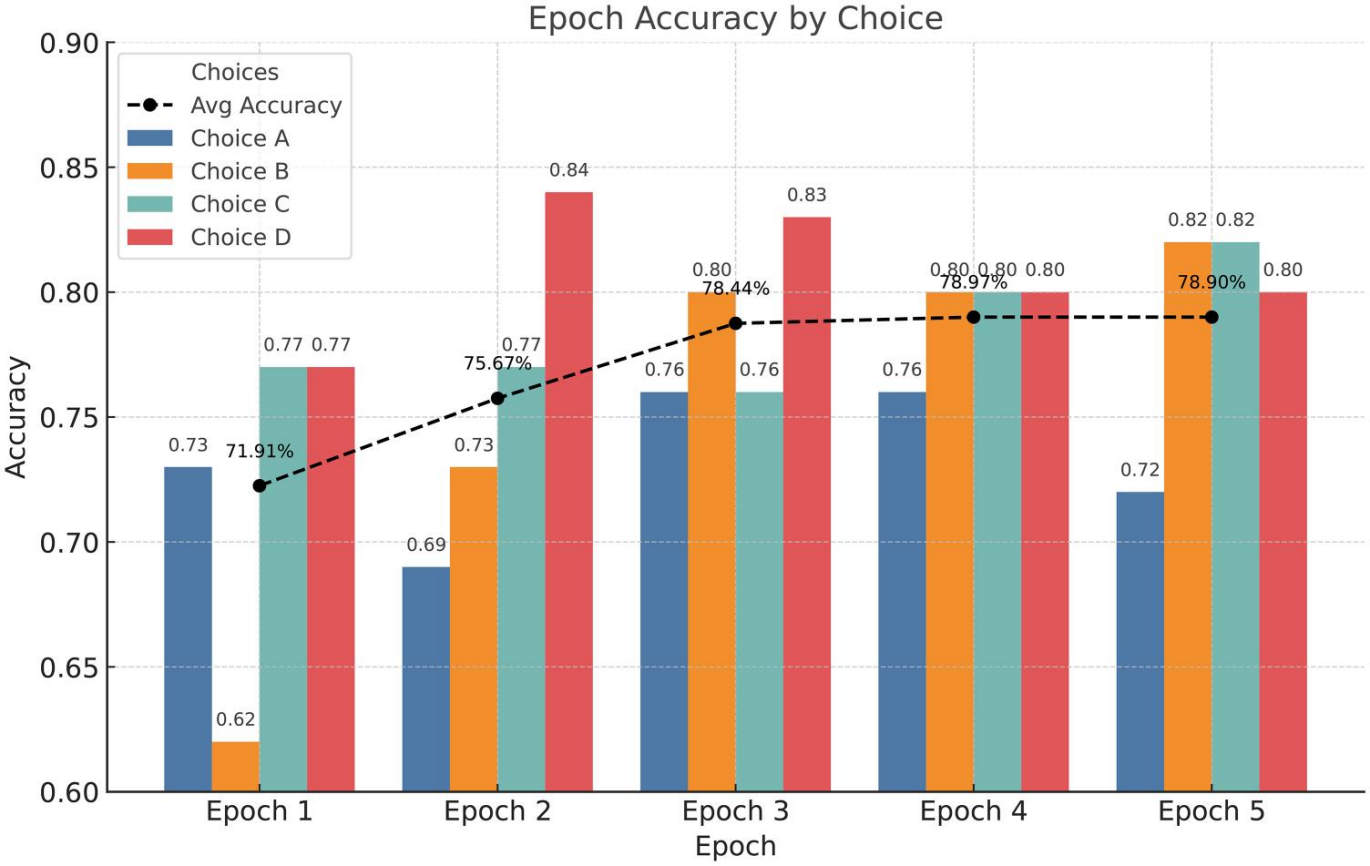
Performance evaluation of a visual question answering model over 5 epochs across different choices (A, B, C, D), along with the model’s average accuracy.

Additionally, Figure 6 presents the model’s average accuracy across epochs when trained on the curated version of the dataset. The accuracy improves steadily across epochs, with the highest performance observed around Epoch 4, reaching an average accuracy of 78.97%. Notably, choices B and C exhibit the most consistent improvements, showcasing their dominance in the dataset distribution.

As part of the study, we investigated various prompt formulations for addressing the VQA task. The corresponding results, detailed in Table 2, were obtained using an imbalanced version of the dataset. This choice was deliberate, as the primary objective at this stage was to discern the optimal prompt structure for the task under consideration.

**Table 2.** Findings from comparing VQA accuracy against other models and input formats, conducted on an imbalanced version of the dataset.

Accuracy of	A	B	C	D	AVG
Vit3D Model^25^	–	–	–	–	0.61
Original answer format^a^	0.56	0.84	0.84	0.78	0.76
X-format^b^	0.55	0.84	0.83	0.75	0.74
“Correct choice is ...”^c^	0.59	0.85	0.83	0.76	0.76
“...”^d^	0.58	0.84	0.83	0.73	0.75

a The original format refers to the format used by the M3D model, which is like "***A. answer choice***".

b X-Format in the table denotes the scenario in which "***A***" was replaced with the letter "***X***".

c Modified answer format where ... refers to letter ***A***, ***B***, ***C*** or ***D***.

d Modified answer format where only the correct choice letter is used.

## Discussion

The integration of AI into medical imaging has revolutionized diagnostic and clinical workflows, but significant challenges remain, particularly when addressing tasks such as MRG and VQA from 3D data. These tasks require models capable of interpreting complex, multimodal data, extracting meaningful insights from volumetric medical data, and aligning them with clinically accurate text.

For MRG, we initially pretrained the 3D medical vision encoder using a CLIP-like approach on the AMOS-MM dataset. However, it became evident that the high degree of similarity in report content, particularly for anatomically similar organs, resulted in model confusion. This confusion likely stemmed from the model’s difficulty in distinguishing subtle variations in text descriptions of similar anatomical structures, reducing the overall accuracy of image-text alignment. Given the significant similarity between text-image pairs, the limited dataset size, and the computational demands, we opted to use the M3D CLIP model. With necessary modifications, we achieved an average GREEN score of 0.409, with consistent performance across the 3 target organs.

For hyperparameter tuning, we systematically explored a range of learning rates, epochs, dataset variants, and temperature settings. Specifically, we tested learning rates of 7e−4, 7e−6, 5e−5, and 5e−4, training the model for 5 epochs across 4 distinct dataset versions (Table 1). Notably, the inclusion of 2000 filtered data points led to an improvement of approximately 5% in chest organ results and enabled the pelvis organ to achieve a GREEN score exceeding 0.50. These results underscore the benefits of combining strategies, as implemented in version 3, to address both dataset imbalance and caption length variability.

In addition, we investigated the effect of temperature settings, ranging from 0.1 to 0.9, to modulate the creativity of the model’s predictions. Lower temperature settings (eg, 0.1) consistently led to higher performance scores (Figure 3), likely because reduced randomness encourages more deterministic and conservative predictions. This trend indicates that moderate temperature values optimize the balance between creativity and consistency, leading to improved performance, particularly in the abdomen and pelvis regions. The sharp decline at higher temperature values suggests increased randomness, which negatively impacts overall accuracy. We hypothesize that this outcome stems from the nature of the pretraining dataset, which includes a significant proportion of reports describing normal or healthy conditions. Lower temperatures appear to align the model’s predictions with these conservative patterns, enhancing medically coherent outputs.

Interestingly, Figure 4A highlights that as the number of matched findings increases, the GREEN score improves, while clinically significant errors decrease. Moreover, Figure 4B reveals that cases with zero significant errors often correspond to shorter predicted token lengths (below 50). While shorter predictions are typically more focused, they may sacrifice comprehensiveness. Longer predictions, on the other hand, increase the number of matched findings but also raise the risk of significant errors. This trade-off demonstrates the challenge of balancing prediction length with accuracy. Furthermore, Figure 4C highlights that when the predicted token length closely aligns with the ground-truth token length, the GREEN score tends to exceed 0.4, reinforcing the importance of length consistency for improved performance. The results indicate that a closer alignment between predicted and ground-truth lengths tends to yield higher GREEN scores, suggesting that token length consistency plays a significant role in report quality.

For VQA, balancing the dataset significantly improved the model’s accuracy. By redistributing answers from choices B and C to underrepresented Class A and D, we equalized the dataset distribution. This restructuring increased the model’s accuracy from 75% to nearly 79%. Figure 5 compares the results of the imbalanced and balanced datasets, showing how balancing mitigated biases. Prior to balancing, the model exhibited a recall of 0.57 for Class B and a precision of 0.6 for Class A, indicative of a bias towards predicting the majority class. Post-balancing, these issues were resolved.

Besides that, while the GREEN score performs well as an evaluation metric, its application to lengthy medical reports presents opportunities for further improvement in real-world deployment. Furthermore, given the detailed and comprehensive nature of full-length radiology reports, mitigating the GREEN score’s inherent bias toward shorter texts could further improve its capacity to accurately assess clinical quality across longer reports.

On the other hand, the ground-truth reports, by their nature, contain a preponderance of sentences describing healthy conditions. As a result, models can achieve high GREEN scores by predominantly predicting healthy outcomes, which reflects the statistical bias in the dataset rather than true generalization performance.

This imbalance in dataset composition further complicates the interpretation of the GREEN score. The metric does not explicitly differentiate between true positives (TP) and true negatives (TN), introducing a critical limitation in its interpretability. For instance, a model that predominantly classifies cases as “healthy” in a dataset with a high proportion of healthy examples may achieve a high GREEN score (a very high number of “Matched Findings”) due to an abundance of TNs, even if its ability to detect abnormalities (TPs) is poor.

TPs correspond to correctly identified cases of disease or abnormality, directly reflecting the model’s diagnostic capability, while TNs represent accurate identification of healthy cases. While both are important, the lack of distinction between these 2 types of correct predictions makes the GREEN score insufficient for evaluating nuanced performance.

Additionally, due to the unstructured nature of the radiology reports in the AMOS-MM dataset, we were unable to conduct a detailed analysis of disease-specific distributions or assess the model’s performance on individual pathologies. This limitation highlights an opportunity for future work to address dataset balance, develop complementary metrics, and pathology-level annotations that can more effectively assess model performance across diverse clinical scenarios.

It is also worth noting that, due to the computationally intensive nature of these tasks, certain avenues for improvement, such as exploring alternative image encoder architectures, increasing input resolution (eg, incorporating more slices), and experimenting with larger language models, remain unexplored. These directions present promising opportunities for future work.

## Conclusion

In summary, this study highlights the promise of multimodal AI systems, exemplified by the Phi3-V pipeline, in addressing the complexities of 3D medical imaging. By integrating visual and textual data, our approach not only enhances the generation of clinically coherent medical reports but also improves performance in VQA tasks. These results underscore the untapped potential of 3D imaging in clinical workflows and lay a foundation for further advancements in automated medical image analysis to support accurate and efficient healthcare decision-making.
